# Markedly Elevated Serum Level of T-Helper Cell 17-Related Cytokines/Chemokines in Acute Myelin Oligodendrocyte Glycoprotein Antibody-Associated Optic Neuritis

**DOI:** 10.3389/fneur.2020.589288

**Published:** 2020-11-13

**Authors:** Hao Kang, Hongyang Li, Nanping Ai, Hongjuan Liu, Quangang Xu, Yong Tao, Shihui Wei

**Affiliations:** ^1^Department of Ophthalmology, Beijing Chaoyang Hospital, Capital Medical University, Beijing, China; ^2^Department of Ophthalmology, Beijing Friendship Hospital, Capital Medical University, Beijing, China; ^3^Department of Ophthalmology, The Chinese People's Liberation Army General Hospital, Beijing, China

**Keywords:** T helper cell 17 (Th17), cytokines, chemokines, optic neuritis, MOG-IgG, AQP4-IgG

## Abstract

**Purpose:** The purpose of this study was to examine the differences in immunopathogenesis based on the cytokine/chemokine profiles in myelin oligodendrocyte glycoprotein antibody (MOG-IgG)-positive and -negative groups.

**Methods:** We measured the levels of T-helper cell 17 (Th17) cell-related cytokines/chemokines in 74 serum samples, which were divided into four groups: healthy control (HC) group (*n* = 15), idiopathic demyelinating optic neuritis (IDON) group (*n* = 20), aquaporin 4 (AQP4)-IgG-positive optic neuritis (ON) group (*n* = 18), and MOG-IgG positive-ON group (*n* = 21). Serum IL17, IL21, IL28, IL31, CXCL1, CXCL2, CCL2, CCL11, CCL20, and LT-α were detected.

**Results:** The serum of the MOG-IgG-positive ON patients showed an obvious elevation of Th17 cell-related cytokines/chemokines compared with that of all the MOG-IgG-negative ON patients. Serum IL17 and IL21 were significantly higher in the ON patients with MOG-IgG positive than in all the other three groups. The serum levels of IL28, IL31, CXCL1, and CCL11 were higher in the ON patients with MOG-IgG positive than in the HC group and the IDON group. The serum concentration of CCL2, CXCL2, and CCL20 in the MOG-IgG-positive and AQP4-IgG-positive group is higher than that of the HC group. No difference in serum LT-α level was found among the four groups. Adjusted multiple regression analyses showed a positive association of IL17 and IL21 levels with the serum concentration of MOG-IgG in the ON patients.

**Conclusion:** The elevated serum level of Th17 cell-related cytokine/chemokines may play an important role in the pathogenesis of MOG-IgG-positive demyelinating ON.

## Introduction

Optic neuritis (ON) is the most common optic neuropathy affecting young adults. ON can occur in isolation, or as the initial symptom of autoimmune-mediated demyelinating diseases, such as multiple sclerosis (MS) or neuromyelitis optica (NMO)/neuromyelitis optica spectrum disorders (NMOSD) (1). In most cases, NMO is caused by autoantibodies to aquaporin 4 (AQP4-IgG) (2, 3), but 10–20% of patients with NMO are negative for AQP4-IgG (4, 5). Recent studies have shown the presence of IgG antibodies to myelin oligodendrocyte glycoprotein antibody (MOG-IgG) in some NMO/NMOSD patients (6, 7). MOG-IgG is pathogenic in human demyelinating diseases, and it is a biomarker of autoimmune ON and longitudinally extensive transverse myelitis (LETM) (8, 9).

MOG-IgG-seropositive patients had some clinical features different from those with AQP4-IgG seropositive (7). In addition, the histopathology of brain and spinal cord lesions of MOG IgG+ patients has been demonstrated to be different from that of AQP4-IgG+ patients (10, 11). MOG-IgG-related disease is now considered as a disease entity in its own right, immunopathogenetically distinct from MS and from AQP4-IgG-related demyelinating diseases.

Cytokines/chemokines are biologically active intercellular messengers having pleiotropic effects on various cell types resulting in immune system activation (12) In the nervous system, cytokines, and chemokines are involved in the regulation of central nerve system (CNS)-immune system interactions that function as neuromodulators and control neurodevelopment, neuroinflammation, and synaptic transmission (13). CD4+ T helper cells can be divided into four major subsets, and Th17 lineage is a recently discovered subset of CD4+ T-helper cells, which can promote tissue inflammation by induction of inflammatory mediators and recruitment of inflammatory cells (14). Th17 cells coordinate local tissue inflammation through the regulation of inflammatory cytokines and chemokines such as IL-17, IL-21, IL-28, CXCL1, CCL2, CXCL2, CCL11, and CCL20 (15). In this study, we evaluated the levels of Th17-related cytokines and chemokines in serum samples from MOG-IgG-seropositive ON, AQP4-IgG-seropositive ON, and idiopathic demyelinating ON (IDON) patients in the acute phase, in order to investigate the differences in immunopathogenesis based on the cytokine/chemokine profiles.

## Materials and Methods

### Patients

In this study, 59 patients with unilateral or bilateral isolated ON were recruited from the Ophthalmology Department of Beijing Chaoyang Hospital of Capital Medical University and the Chinese People's Liberation Army General Hospital (PLAGH). Recruitment was completed between April 2017 and July 2018. ON was the first symptom in all the patients who fulfilled the diagnosis criteria of ON. All the subjects were treated with methylprednisolone according to the suggestion with ONTT (16). If a minimal response to the corticosteroid therapy and the vision remained below 0.1 were clinically observed, the patient involved was given a total of three to five plasma exchanges.

All blood samples were collected during the acute phase of the disease or within a month of exacerbation. Seventy-four serum samples were drawn from the patients with acute demyelinating ON and from the controls, including 15 samples from the healthy control (HC) group, 20 samples from the IDON group, 18 samples from the AQP4-IgG-seropositive ON group, and 21 samples from the MOG-IgG-seropositive ON group.

Ophthalmic examinations including best-corrected visual acuity (BCVA), intraocular pressure, slit lamp examination, pupillary reactions in unilateral or bilateral asymmetric conditions, and ocular fundus examinations were conducted by professional ophthalmologists.

BCVA was tested by using a Snellen chart and was transformed into logarithm of the minimum angel of resolution (logMAR) values by using Petzold's et al. (17) VA conversion method. If a VA was below 0.01, finger-counting (FC), hand motion (HM), perception of light (LP), and no perception of light (NLP) were tested, and the results were documented accordingly. All patients underwent visual field, electrodiagnostic tests, and orbit and brain magnetic resonance imaging (MRI) examination.

### Aquaporin 4 IgG and Myelin Oligodendrocyte Glycoprotein Antibody Testing

All serum samples were analyzed for the presence of AQP4-IgG by an extracellular live cell-staining immunofluorescence technique using transiently transfected AQP4-expressing cells as previously described (18). Samples were scored as positive or negative by at least two independent experiments. A dilution of 1:1,000 was employed as the maximum positive value and 1:10 as the cut-off for positive and negative cases. MOG-IgG detection was performed by CBA with full-length human MOG-transfected HEK293 cells. MOG-IgG titers of ≥1:10 were classified as positive.

### Cytokine and Chemokine Assay

Th17-related cytokines (IL-17, IL-21, IL-28, and IL-31) and chemokines (CXCL1/GRO alpha, CXCL2/GRO beta, CCL2/MCP-1, CCL20/MIP-3, and CCL11/eotaxin) and LT-alpha/TNF-beta were measured by means of ELISA. The regression equation of the standard curve (*R*^2^ > 0.98) was calculated on the basis of the standard concentration and the corresponding *A*-value. Similarly, the corresponding sample concentration was calculated with reference to the sample's *A*-value.

### Ethics Statement

This study was approved by the Ethics Committee of Beijing Chaoyang Hospital and PLAGH, and was conducted following the Declaration of Helsinki in its currently applicable version. Written informed consents were obtained from each patient.

### Statistical Analysis

Statistical analysis was performed by using SPSS for Windows, Version 21.0. Continuous variables were analyzed using a nonparametric test (Mann–Whitney U test). The Chi-squared test, or Fisher's exact test if applicable, was used to analyze the categorical data. The differences among any three groups were identified by using the ANOVA or Kruskal–Wallis test. In order to reduce type I errors, Bonferroni correction had been applied on the *P*-values. Correlation ranks were evaluated by Spearman's rank correlation tests.

## Results

### Demographic Data and Clinical Characteristics

The demographic data and clinical characteristics of all the 59 Chinese ON patients were compared (Table 1). The mean age at disease onset was similar in the three groups. Female predominance was apparent in the IDON patients (82.4%) and the AQP4-IgG seropositive-ON patient (94.1%), but the MOG-IgG-seropositive ON patients were mostly male, with a male-to-female ratio of 11:10. Compared to the IDON group (10/20, 50.0%), the AQP4-IgG-seropositive ON (15/18, 83.3%) and MOG-IgG-seropositive ON (17/21, 81.0%) patients were more likely to have accompanying ocular pain. The proportion of disc swelling and monocular/binocular involvement showed no difference between the three groups. Recurrent ON appeared frequently among all the AQP4-IgG-seropositive ON (18/18, 100.0%) and MOG-IgG-seropositive ON patients (21/21, 100.0%), and the IDON patients (8/20, 60%) had a more frequent monophasic course. In the routine CSF analysis, no significant difference in the median CSF white cell count (no/mm^3^) and the CSF IgG level (mg/L) between the three groups was found. CSF total protein (mg/L) was significantly higher in the AQP4-IgG-seropositive ON group than that in the other two groups (*P* < 0.001; AQP4-IgG + ON vs. MOG-IgG + ON: *P* = 0.003, AQP4-IgG + ON vs. IDON: *P* = 0.001). There were no significant differences between the three groups in the proportion of optic lesion in MRI. None of the patients in the three groups had MRI results that met the radiological diagnostic criteria of MS or NMO. VA at first attacks in the acute phase and VA recovery at the last follow-up were compared in the AQP4-IgG-seropositive ON, MOG-IgG-seropositive ON, and IDON patients. No differences in visual loss during the acute stage were observed between the three groups. At the last follow-up, the AQP4-IgG-seropositive ON patients were significantly more likely to get poor VA recovery over time than the other patients (*P* = 0.015; AQP4-IgG + ON vs. MOG-IgG + ON: *P* = 0.012, AQP4-IgG + ON vs. IDON: *P* = 0.272).

**Table 1:** Epidemiologic and disease characteristics of ON patients and healthy controls.

	**IDON**	**AQP4 + ON**	**MOG + ON**	**HC**	***P*-value**
Number of patients	20	18	21	15	–
Age at onset (years)	34.50 ± 12.96	37.06 ± 12.95	35.81 ± 14.08	35.20 ± 12.39	0.945
Gender (male:female)	3:17	1:17	11:10	4:11	**0.005**
Ocular pain (*n*, %)	10 (50.0)	15 (83.3)	17 (81.0)	–	**0.036**
Disc swelling (*n*, %)	8 (40.0)	7 (38.9)	7 (33.3)	–	0.894
Bilateral, ever (%)	5 (25.0)	7 (38.9)	9 (42.9)	–	0.461
Recurrent ON onset (*n*, %)	8 (40.0)	18 (100.0)	21 (100.0)	–	**<0.001**
Median CSF white cell count (*no*./mm^3^)	2.10 ± 4.12	6.89 ± 11.29	3.00 ± 3.67	–	0.095
CSF protein (mg/L)	288.82 ± 85.01	422.53 ± 132.88	304.42 ± 94.03	–	**<0.001**
CSF IgG level (mg/L)	2.48 ± 0.96	3.55 ± 3.02	2.14 ± 0.64	–	0.05
BCVA at first ON attacks in acute time (logMAR)	1.85 ± 0.89	2.17 ± 0.79	1.82 ± 0.81	–	0.369
BCVA recovery at last follow-up (logMAR)	0.64 ± 0.67	1.00 ± 0.78	0.37 ± 0.51	–	**0.015**

*ON, optic neuritis; AQP4: aquaporin-4; MOG, myelin oligodendrocyte glycoprotein; IDON, idiopathic demyelinated optic neuritis; HC, healthy controls; CSF, cerebrospinal fluid; MRI: magnetic resonance imaging; BCVA, best-corrected visual acuity. The bold values are used to indicate values with P < 0.05*.

### Comparison of T-Helper Cell 17-Related Serum Cytokine/Chemokine Levels Between the Myelin Oligodendrocyte Glycoprotein Antibody-Seropositive Optic Neuritis, Aquaporin 4-IgG-Seropositive Optic Neuritis, Idiopathic Demyelinating Optic Neuritis Patients, and the Healthy Controls

**Figure 1:**
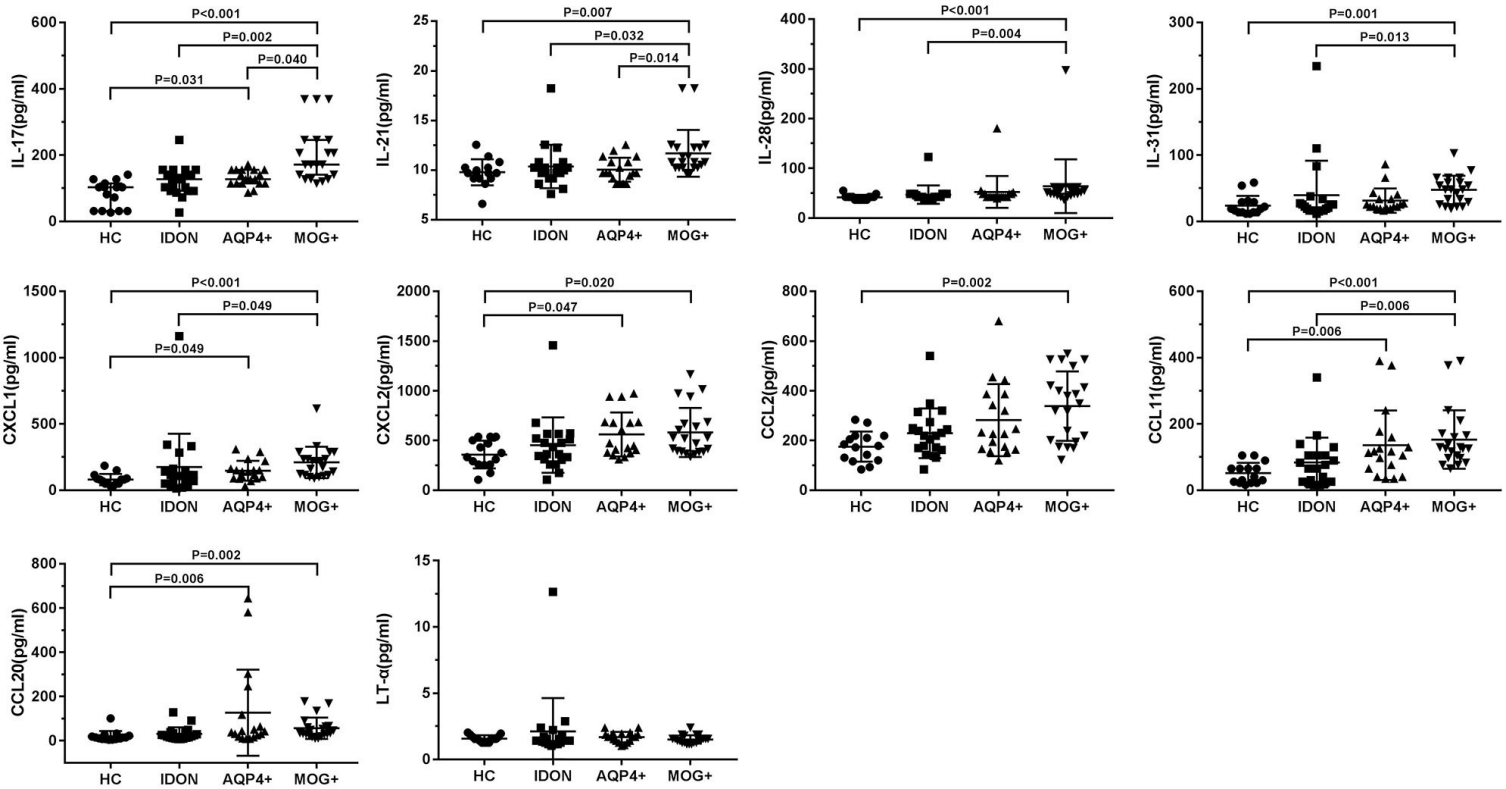
Comparison of T-helper cell 17 (Th17)-related serum cytokine/chemokine levels between myelin oligodendrocyte glycoprotein antibody (MOG-IgG) + optic neuritis (ON), aquaporin 4 (AQP4-IgG) + ON, idiopathic demyelinating optic neuritis (IDON), and healthy controls.

The dot plots of individual serum cytokines/chemokines levels are shown in Figure 1, and the values are summarized in Table 2. IL-17, IL-21, IL-28, IL-31, CXCL1, CXCL2, CCL2, CCL20, and CCL11 were significantly elevated in the MOG-IgG-seropositive ON patients than in the MOG-IgG-patients. The mean concentration of IL-17 in the patients with MOG-IgG-seropositive ON was much higher than the other three groups of patients. Moreover, it was also higher in the patients with AQP4-IgG-seropositive ON than in the HC group (IL-17: AQP4-IgG + ON vs. HC: *P* = 0.031). The concentration of IL-21 in the patients with MOG-IgG-seropositive ON was also higher than in the AQP4-IgG-seropositive ON, IDON, and HC groups. The IL-23 concentration in the patients with MOG-IgG-seropositive ON was also higher than in the IDON and HC groups. The MOG-IgG-seropositive ON patients showed a significantly higher IL-31 level than the IDON and HC patients. The serum CXCL1 and CXCL2 concentration in the patients with MOG-IgG-seropositive ON and AQP4-IgG-seropositive ON was also higher than that in the HC group (CXCL2: AQP4-IgG+ ON vs. HC: *P* = 0.047). The MOG-IgG-seropositive ON patients had a significantly higher CCL2 level than the HC patients. In the serum concentration of CCL20, the MOG-IgG-seropositive ON and AQP4-IgG-seropositive ON patients showed higher levels than the HC group (CCL20: AQP4-IgG + ON vs. HC: *P* = 0.006). CCL11 was significantly elevated in the two autoantibody-associated ON patients than in the HC group (CCL11: AQP4-IgG + ON vs. HC: *P* = 0.006). The CCL11 concentration in the MOG-IgG-seropositive ON patients was also higher than that in the IDON group. No significant difference was found in the serum concentration of LT-α between the four groups.

**Table 2:** Serum level of Th17-related cytokine/chemokines of optic neuritis patients.

	**MOG-IgG + ON (*n* = 21)**	**AQP4-IgG + ON (*n* = 18)**	**IDON (*n* = 20)**	**HC (*n* = 15)**	**P^†^**	**P1^§^**	**P2^§^**	**P3^§^**
IL-17 (pg/ml)	231.58 ± 156.74	131.33 ± 24.02	122.07 ± 44.56	81.73 ± 41.19	**<0.001**	**0.040**	**0.002**	**<0.001**
IL-21 (pg/ml)	11.70 ± 2.35	10.05 ± 1.20	10.38 ± 2.19	9.79 ± 1.32	**0.002**	**0.014**	**0.032**	**0.007**
IL-28 (pg/ml)	63.99 ± 54.00	52.49 ± 32.16	46.98 ± 18.46	41.34 ± 5.23	**<0.001**	0.081	**0.004**	**<0.001**
IL-31 (pg/ml)	47.51 ± 21.48	31.27 ± 18.28	39.61 ± 51.85	23.79 ± 14.67	**0.001**	0.167	**0.013**	**0.001**
CXCL1 (pg/ml)	209.71 ± 119.21	146.49 ± 74.65	175.12 ± 251.10	80.06 ± 42.96	**<0.001**	0.652	**0.049**	**<0.001**
CXCL2 (pg/ml)	581.23 ± 246.09	560.92 ± 221.33	453.84 ± 278.00	358.50 ± 139.63	**0.008**	>0.99	0.245	**0.020**
CCL2 (pg/ml)	56.44 ± 48.47	126.48 ± 194.93	30.52 ± 30.08	175.39 ± 60.81	**0.003**	>0.99	0.126	**0.002**
CCL20 (pg/ml)	2.31 ± 0.12	1.17 ± 0.21	1.66 ± 0.13	19.84 ± 23.87	**0.001**	>0.99	0.131	**0.002**
CCL11 (pg/ml)	152.83 ± 88.16	135.75 ± 104.86	83.41 ± 75.38	21.34 ± 31.11	**<0.001**	>0.99	**0.006**	**<0.001**
LT-α (pg/ml)	1.51 ± 0.31	1.68 ± 0.39	2.11 ± 2.52	1.57 ± 0.25	0.42	–	–	–

*Th17, T-helper cell 17; ON, optic neuritis; AQP4, aquaporin 4; MOG, myelin oligodendrocyte glycoprotein; IDON, idiopathic demyelinated optic neuritis; HC, healthy controls; P1, MOG-IgG + ON and AQP4-IgG + ON; P2, MOG-IgG + ON and IDON; P3, MOG-IgG + ON and HC*;

† *Kruskal–Wallis H-test*;

§ *Bonferroni method*.

*The bold values are used to indicate values with P < 0.05*.

### Relationship Between T-Helper Cell 17-Related Serum Cytokines/Chemokines and Serum Myelin Oligodendrocyte Glycoprotein Antibody Titer in the Myelin Oligodendrocyte Glycoprotein Antibody-Seropositive Optic Neuritic Patients

**Figure 2:**
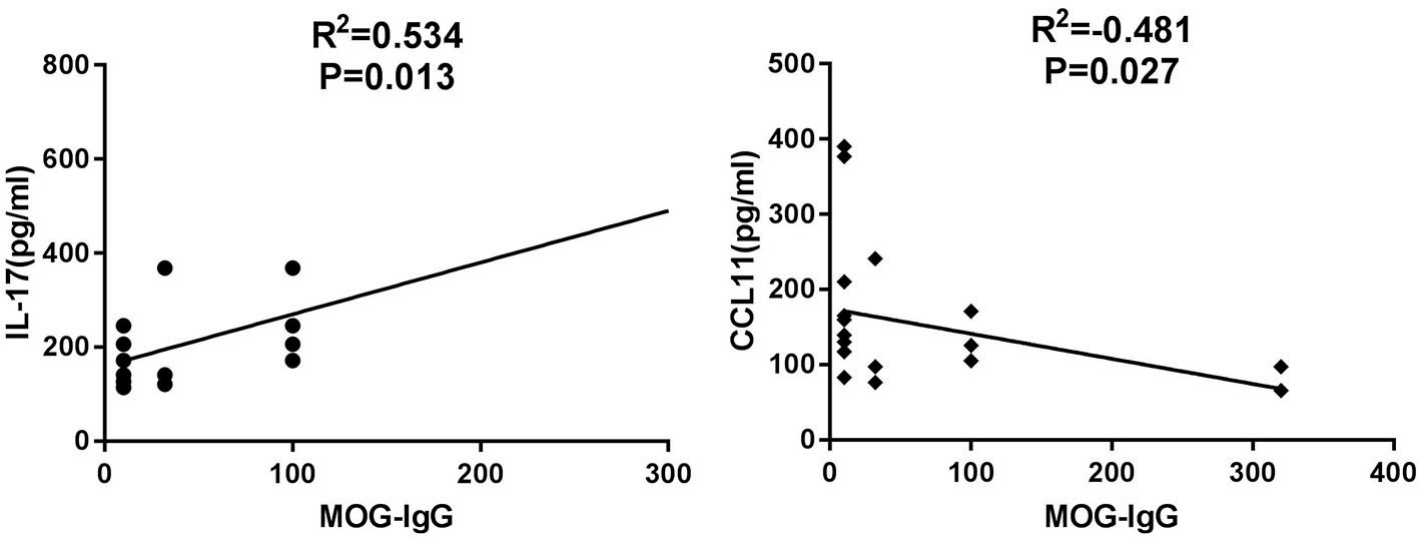
Relationship between Th17-related serum cytokines/chemokines and serum MOG-IgG titer in MOG-IgG-seropositive ON patients.

We then analyzed the potential correlations between the elevated Th17-related cytokines/chemokines levels and the titer of MOG-IgG in 21 MOG-IgG-seropositive ON patients (Table 3). Correlation analyses showed that serum IL-17 was positively correlated with the titer of MOG-IgG in the patients' serum (*r* = 0.534, *P* = 0.013; Figure 2). The serum concentration of CCL11 was negatively correlated with the titer of MOG-IgG in the MOG-IgG-seropositive ON patients (*r* = −0.481, *P* = 0.027; Figure 2). However, no significant correlation between the other cytokines/chemokines and the serum titer of MOG-IgG was observed.

**Table 3:** Spearman's correlation coefficient (r) of the association between serum cytokines/chemokines and MOG-IgG titer in 21 MOG-IgG-seropositive ON patients.

	**MOG IgG (*n* = 21)**	**Correlation coefficient**	***P***
IL-17 (pg/ml)		**0.534**	0.013^*^
IL-21 (pg/ml)		0.163	0.479
IL-28 (pg/ml)		−0.104	0.653
IL-31 (pg/ml)		−0.195	0.397
CXCL1 (pg/ml)		−0.218	0.344
CXCL2 (pg/ml)		0.280	0.220
CCL2 (pg/ml)		−0.074	0.751
CCL20 (pg/ml)		0.176	0.445
CCL11 (pg/ml)		**−0.481**	0.027^*^
LT-α (pg/ml)		−0.187	0.416

*ON, optic neuritis; MOG, myelin oligodendrocyte glycoprotein. The bold values are used to indicate values with P < 0.05*.

* *P < 0.05*.

## Discussion

In the present study, we compared the Th17-related serum cytokines/chemokines in healthy adults and ON patients with different etiologies. In the MOG-IgG-seropositive ON patients, we noted a significant upregulation of Th17 cell-related serum cytokines/chemokines. These data are in agreement with previous studies of Th17 changes in other autoimmune diseases, as Th17 cells are a key stakeholder in the pathogenesis of many autoimmune disorders (15).

Th17 cells have been involved in several autoimmune disorders, and they seem to be relevant in CNS autoimmunity development. Th17-related molecules have also been shown to correlate with parameters of disease activity and severity in CNS inflammatory demyelinating diseases (19). IL-17 is a proinflammatory cytokine that upregulates the expression of inflammatory genes. More importantly, elevated IL-17 levels have been observed in autoimmune diseases like MS, inflammatory bowel disease, psoriasis, and rheumatoid arthritis (20). Although IL-17 is the signature cytokine of Th17 cells, many studies have shown that other cytokines related with Th17 cells are also significant in the pathogenesis of inflammatory responses. The release of IL-6 and IL-21 by polyclonally activated CD4+ T cells obtained from NMO patients was shown to have direct correlations with neurological disability (14). IL-31 is also produced mainly by CD4+ T cells. A recent report has shown that the serum concentration of IL-31 significantly increased in NMOSD patients and positively correlated with the serum level of IL-17 in those patients (21). Moreover, Th17 cells necessitate a large quantity of chemokines and chemokine receptors to cross the blood–brain barrier (BBB), which enables them to disrupt the BBB and access the CNS through some different pathways. IL-17 is a key factor in the disruption of the BBB by directly impairing its integrity (22). *In vitro* and *in vivo* studies have shown that through the action of IL-17, Th17 cells can efficiently break down BBB tight junctions, bring out high levels of the cytolytic enzyme granzyme B, and provide impetus to the recruitment of additional CD4+ lymphocytes from the systemic circulation into the CNS (23). In addition, Th17 cells are also capable of inducing CXCL1 and CXCL2, chemokines that are powerful attractants for polymorphonuclear cells, and of contributing much to the disruption of the BBB in experimental autoimmune encephalomyelitis (EAE) (24).

NMOSD is a severe CNS autoimmune inflammatory disorder, which has always been recognized as a B-cell-mediated humoral immune disease. However, B-cell depletion therapy was not efficacious in some NMOSD patients, including both antibody-seropositive or -seronegative patients (25). One possible reason is that B cell-mediated immunity may not be the sole contributor to NMOSD-like lesions, and other components like CD4+ T cells, especially Th17 cells, may also play potential roles. Studies have demonstrated that T-cell-mediated immunity may participate in the pathological process of NMOSD, especially in the Th17 phenotype (26–28). Another possible explanation is that MOG-IgG-related demyelination in the optic nerve and the spinal cord can partly explain AQP4-IgG-seronegative NMOSD patients. In this study, the results showed that both AQP4-IgG-seropositive ON and MOG-IgG-seropositive ON have increased the serum concentration of Th17-related cytokines and chemokines compared with that in the control subjects, while the change in MOG-IgG-seropositive ON is more significant. This result supported the hypothesis that Th17 cells are highly activated in MOG-IgG-seropositive ON patients during the disease's acute exacerbation and relapse stages. From a clinical point of view, the difference in cytokines and chemokines between different types of ON suggests its value in the differential diagnosis of disease, especially in the early stage of diseases.

MOG-IgG has been detected in a proportion of AQP4-IgG-seronegative NMOSD patients. MOG-IgG is a biomarker for patients with CNS demyelinating diseases that have distinct demographic, serologic, clinical, and radiologic features from classical MS and from AQP4-IgG-mediated NMOSD, suggesting that MOG-IgG might mediate a distinct disease (29). Moreover, the histopathology of the lesions of MOG-IgG-seropositive patients has been shown to differ from that of AQP4-IgG-mediated CNS lesions (10, 11). In a retrospective multicenter study of MOG-IgG-seropositive NMOSD patients, 88% of the patients developed acute ON at least once, 56% of the patients developed acute myelitis at least once, 44% of the patients only had a history of ON but not of myelitis, while only 12% of the patients had a history of myelitis but not of ON (29). This is consistent with another study that demonstrated that the optic nerve is more susceptible than the spinal cord in MOG-IgG-related CNS autoimmunity (30). A recent study has shown that autoimmune inflammatory infiltrates in the optic nerve are different from inflammation in other parts of the CNS, suggesting that the optic nerve might be an immunologic compartment different from the spinal cord. Compared with the spinal cord, Th17 cells prevailed in the optic nerve and the brain. Local tissue expression of IL-17 was the highest in the optic nerve, suggesting that Th17-related immunopathology was dominant in the optic nerve. The study concluded that the optic nerve compartment is particularly prone to supporting IL-17-mediated inflammatory immune responses during CNS autoimmunity, and neutralization of IL-17 is sufficient to prevent structural damage to the optic nerve (31). In the present study, the MOG-IgG-seropositive ON patients had a higher concentration of Th17-related cytokines and chemokines than the other types of ON patients. Furthermore, the serum level of IL 17 in the acute phase of the MOG-IgG-seropositive ON was positively correlated with serum MOG-IgG titer, indicating that a higher serum level of IL 17 during the acute phase was related to the MOG-IgG induced. The results of this study have further confirmed that MOG-IgG-related neuroinflammation is immunopathogenetically distinct from classical MS and AQP4-IgG-induced demyelinating disorders. More importantly, these results highlight the important role of the Th17 cells for neuronal demyelination in MOG-IgG-induced neuroinflammation.

There are some limitations in this study. First, the number of ON patients enrolled in the study was not sufficient, and no follow-up after treatment had been done. Second, we only detected the serum level of the cytokines and chemokines. It would have been much better if we had detected both the CSF and serum levels simultaneously, which could contribute to probing the underlying mechanisms of Th17 cells in MOG-IgG-related demyelinating diseases. Third, we did not compare the serum levels of the cytokines and chemokines of the same patient during the different stages of the disease. Long-term prospective multicenter studies are required to analyze the detailed immunopathologic mechanisms of Th17 cells in different types of demyelinating ON.

In conclusion, this study suggests that Th17 cells were highly activated in MOG-IgG-seropositive ON patients with significantly increased serum cytokines and chemokines. This cytokine/chemokine profiling provides new insights into ON pathogenesis and is useful in monitoring disease activity. Further research is required to clarify if interference in the Th17 pathway can reduce inflammation in the CNS during disease onset and relapses.

## Data Availability Statement

All datasets presented in this study are included in the article/supplementary material.

## Ethics Statement

The studies involving human participants were reviewed and approved by Ethics Committee of the Chinese People's Liberation Army General Hospital and Beijing Chaoyang Hospital of Capital Medical University. The patients/participants provided their written informed consent to participate in this study.

## Author Contributions

HK, SW, and YT designed and conducted the study. HK, NA, HLiu, and QX collected, managed, analyzed, and interpreted the data. HK and HLi prepared the manuscript. All authors reviewed and made the final approval of the manuscript.

## Conflict of Interest

The authors declare that the research was conducted in the absence of any commercial or financial relationships that could be construed as a potential conflict of interest.
